# A Dissertation on Osteo-Arthritis

**Published:** 1893-09

**Authors:** 


*A Dissertation on Osteo-Arthritis.* By W. H. RUSSELL FORSBROOK, M.D. London: H. K. LEWIS. 1893.

During the past five years rheumatoid arthritis has been "in the air," and is a favourite subject for academic theses. Such theses do not pretend to convey the results of much original research; but they are useful as compilations, and really often determine the future studies of a frank and open mind. Dr. Forsbrook's volume is a good example of the "thesis" style, although here and there rather dogmatic for an early student in a difficult subject. The way is, however, being cleared by the work of many independent observers; and the English doctrine will soon be proved beyond all challenge that rheumatoid arthritis is essentially and emphatically a neural disease.

Of the many titles which have been conferred on this disease, rheumatoid arthritis is certainly by a long way the best. Rheumatic arthritis is decidedly bad, and rheumatic gout the worst of all. Osteo-arthritis has the advantage of official recognition, but is not understood by the laity: and it is *very* important for the laity to distinguish this malady from its counterfeits. But first, it must be decisively recognised by medical men themselves. Two splendid opportunities of educating junior practitioners and senior students have been virtually thrown away. The writer on this subject in Quain's *Dictionary*, though a well-known physician, gives a sketch which is not only incomplete, but absolutely misleading; and the chapter in Dr. Kingston Fowler's *Dictionary* is far from satisfactory. Dr. Archibald Garrod's book is an excellent summary of existing knowledge; but its literary style has little force or vividness, and therefore lacks teaching power.

A short and well-written treatise, on post-graduate lines, would fill a void in the literature of rheumatoid arthritis. Cut out the usual archaic padding about Haygarth and Heberden, except a short paragraph on the principle of the "commemoration of benefactors." Let there be a sharply-cut definition of the disease; strip away the parasitic nomenclature which obscures its identity, and draw the clinical outlines with precision and brevity. Mark and learn the early distinctive points —the "connoting signs" of our neighbour, Dr. Spender. When present these signs are of demonstrable value, and determine an otherwise doubtful case. Trace the etiology, the neural complications, the atrophic phenomena, and the rarer collateral symptoms and affinities. And when all these facts are duly impressed upon the medical imagination, the treatment will be logically established; and the common scandal of therapeutically looking at rheumatoid arthritis as if it were gout will be taken away.

A little while ago we went round the Bath Mineral Water Hospital with one of the medical staff. Outside the Salpêtrière at Paris there is probably no similar home of living pathology; and we only wish that we could annex it for a time to our medical school, and use it for bedside instruction in articular diseases.

